# The Plant Circadian Oscillator

**DOI:** 10.3390/biology8010014

**Published:** 2019-03-12

**Authors:** C. Robertson McClung

**Affiliations:** Department of Biological Sciences, Dartmouth College, Hanover, NH 03755, USA; c.robertson.mcclung@dartmouth.edu; Tel.: +1-603-646-3940

**Keywords:** circadian rhythms, circadian clock, transcriptional feedback loops, plant circadian clock, posttranscriptional, posttranslational, alternative splicing, protein stability

## Abstract

It has been nearly 300 years since the first scientific demonstration of a self-sustaining circadian clock in plants. It has become clear that plants are richly rhythmic, and many aspects of plant biology, including photosynthetic light harvesting and carbon assimilation, resistance to abiotic stresses, pathogens, and pests, photoperiodic flower induction, petal movement, and floral fragrance emission, exhibit circadian rhythmicity in one or more plant species. Much experimental effort, primarily, but not exclusively in *Arabidopsis thaliana*, has been expended to characterize and understand the plant circadian oscillator, which has been revealed to be a highly complex network of interlocked transcriptional feedback loops. In addition, the plant circadian oscillator has employed a panoply of post-transcriptional regulatory mechanisms, including alternative splicing, adjustable rates of translation, and regulated protein activity and stability. This review focuses on our present understanding of the regulatory network that comprises the plant circadian oscillator. The complexity of this oscillatory network facilitates the maintenance of robust rhythmicity in response to environmental extremes and permits nuanced control of multiple clock outputs. Consistent with this view, the clock is emerging as a target of domestication and presents multiple targets for targeted breeding to improve crop performance.

## 1. Introduction

This special issue celebrates the 2017 Nobel Prize in Physiology awarded to Jeff Hall, Michael Rosbash, and Mike Young. Their work on circadian rhythms in *Drosophila* established the molecular basis underlying circadian rhythms as a negative feedback loop, based on transcription and translation, by which a positive regulator (activator), consisting of a heterodimer of CLOCK and CYCLE, activates expression of a repressor, a second heterodimer of PERIOD and TIMELESS, of its own expression [1]. Although the story is complicated, with elements of post-translational regulation and additional interlocked feedback loops [2,3,4], that fairly simple yet profound insight of a transcriptional feedback loop can be applied to the circadian clocks of most, if not all, eukaryotes.

Historically, circadian rhythms were not first observed in Drosophila but rather in plants. The first description of a diurnal rhythm was in the fourth century BC, when Androsthenes described sleep movements of tree leaves during the expeditions of Alexander the Great [5], although there was no indication that these rhythms were recognized as endogenous in origin. It was not until 1729 that de Mairan [6] established that the rhythmic leaf movements in Mimosa persisted in constant conditions and, hence, were endogenous. A century later, de Candolle [7] determined that the periodicity of rhythmic leaf movements on *Mimosa pudica* was only approximately 24 h, making these rhythms “circadian.” He also showed that these rhythms could be inverted by reversing the light-dark cycle, thereby demonstrating entrainment. Over the remainder of the 19th century, these observations were extended to many plant species by a number of scientists, including Charles Darwin [8]. Temperature compensation was first established by Bünning [9], who showed that the Q_10_ of the period of leaf movement in *Phaseolus coccineus* was only 1.2, much less than the Q_10_ of ~2 exhibited by a typical chemical reaction. Thus, research investigating rhythmic leaf movements of plants defined what are now accepted as the essential characteristics of circadian rhythms: Endogenous origin, period of approximately 24 h, entrainment to the environment, and temperature compensation. It also became clear that many more plant processes, including germination, growth, gas exchange, photosynthesis, floral scent emission, and flower opening, exhibited circadian rhythms [10]. More detailed historical accounts of the early investigations into plant circadian rhythms are available [11,12].

The modern era of investigation into the molecular basis for plant circadian rhythms began with the observation that the transcript abundance of three photosynthetic genes of pea, including that encoding a light-harvesting chlorophyll *a/b* binding protein (*LHCB*, also called *CAB*), cycled with a circadian period [13]. The transcription rate for a wheat *CAB* gene was subsequently shown to be under circadian control [14]. It is now well-established that plant circadian clocks orchestrate pervasive transcriptional reprogramming on a daily basis. Roughly 1/3 of transcripts in Arabidopsis show circadian rhythms [15,16,17], although the proportion of the transcriptome that cycles in abundance increases substantially under a variety of environmental cycles [18]. Similar broad circadian control of the transcriptome has been observed in other plants, including poplar, *Brassica rapa*, and rice [18,19,20,21].

The identification of behavioral mutants with defects in circadian rhythms, and the identification of the genes responsible for the mutant clock phenotypes, were crucial in elucidating the circadian clock mechanism in Drosophila, beginning with the *period* gene [22,23,24,25,26]. Forward genetic screening, on the basis of altered rhythmic expression of a firefly *LUCIFERASE* (*LUC*) transgene driven by the clock-regulated *LHCB1.1* (also called *CAB2*) promoter yielded the first circadian clock mutant in *Arabidopsis*, which was ultimately shown to carry a loss of function allele of the *TIMING OF CAB2 EXPRESSION1* (*TOC1*) gene [27]. The loss of TOC1 function shortened circadian period in both LUC activity and leaf movement by about 3.5 h, from ~24.5 in the isogenic wild type parent to ~21 h in *toc1-1*. The cloning of *TOC1* identified it as encoding a nuclear protein with sequence motifs similar to those found in two-component signal-transduction systems common in bacteria [28]. This was the first molecular indication that the plant clock might share the design logic of animal and fungal clocks as interlocked feedback loops but is composed of components distinct from those found in animal and fungal clocks.

In the nearly quarter century since the identification of the *toc1-1* mutant, enormous progress has been made towards the illumination of the plant circadian oscillator. It is this progress, and our current vision of the oscillator mechanism, that are the focus of this review.

## 2. The Plant Clock Consists of Multiple Interlocked Transcriptional Feedback Loops

### 2.1. Transcriptional Repression

**Figure 1:**
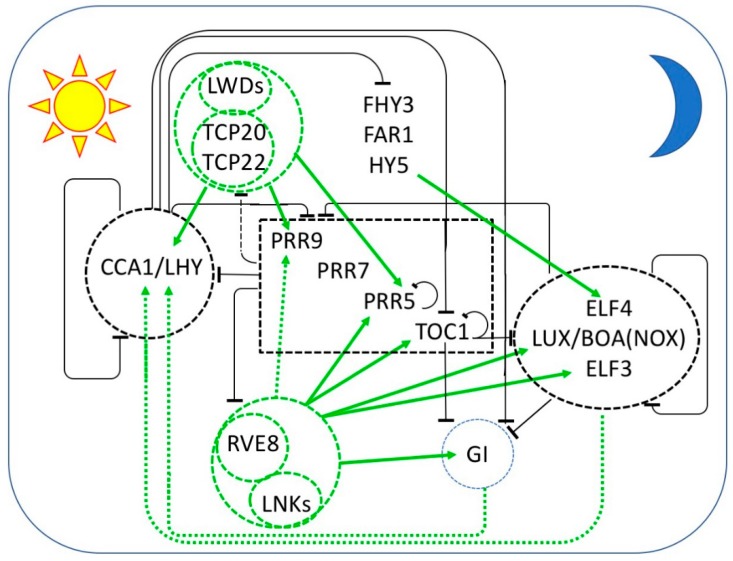
Multiple interlocked transcriptional feedback loops form the core of the circadian oscillator in *Arabidopsis thaliana*. The sequential expression of each component throughout the day/night cycle is shown from left to right, with the morning represented by the sun at the left and the night represented by the moon at the right. Black bars indicate repression and green arrows indicate activation of transcription. Protein complexes are enclosed by dashed lines. Transcriptional repressors are in black and repression is indicated by black bars. Transcriptional activators are in green and transcriptional activation is indicated by green arrows. Dashed arrows indicate relationships that are not established to be direct. At dawn, CCA1 and LHY repress the expression of the *PRR* genes, *TOC1*, *GI*, and the EC members *LUX*, *ELF3,* and *ELF4*. *PRR9*, *PRR7*, *PRR5*, and *TOC1* are sequentially expressed and repress the transcription of *CCA1* and *LHY*, as well as their own transcription. LWD1 and LWD2 are transcriptional co-activators recruited to DNA by TCP20 and TCP22 to promote the expression of *CCA1*, *PRR9*, *PRR7*, and *TOC1*. In the afternoon, transcriptional activation is mediated by the LNKs, transcriptional coactivators recruited to DNA by RVE8 (and probably RVE4 and RVE6). RVE-LNK complexes promote transcription of *PRR9*, *PRR5*, *TOC1 GI, LUX,* and *ELF4*. Additional transcriptional activation of *ELF4* is provided by FHY3, FAR1, and HY5. In the evening, TOC1 represses all of the daytime components as well as *GI*, *LUX*, and *ELF4*. LUX and ELF4 together with ELF3 form the evening complex (EC) which is a transcriptional repressor of *GI, PRR9,* and *PRR7*. GI and an EC variant containing BOA (NOX) seem to be required for the transcriptional activation of *CCA1* and *LHY*.

Most of our knowledge of the plant circadian clock has been learned through forward and reverse genetics and molecular biological approaches in the model plant *Arabidopsis thaliana*. The *Arabidopsis* clock features a remarkably large number of transcription factors arranged in a surprisingly large number of feedback loops (Figure 1). The initial loop consisted of a pair of dawn expressed MYB-related transcription factors, LATE ELONGATED HYPOCOTYL (LHY) and CIRCADIAN CLOCK ASSOCIATED 1 (CCA1) and the evening expressed TOC1. CCA1 and LHY homo- and hetero-dimerize [29,30] and bind to a motif termed the evening element (EE) [15,31]. CCA1 and LHY function as transcriptional repressors of each other, of *TOC1*, and of a large number of targets that includes both central clock genes and multiple output genes [31]. TOC1 similarly functions as a transcriptional repressor [32,33]. Thus, at its core, the *Arabidopsis* clock consists of a negative feedback loop in which multiple morning and evening oscillator components act as reciprocal repressors.

*TOC1* was the first identified of a family of *PSEUDO-RESPONSE REGULATOR* (*PRR*) genes encoding a series of sequentially expressed transcriptional repressors [33,34,35,36,37,38]. *PRR9* and *PRR7* are direct transcriptional targets repressed by CCA1 and LHY [39,40]. PRR9 and PRR7 together with the later-expressed PRR5 repress *CCA1* and *LHY* [36]. TOC1 interacts with CCA1 HIKING EXPEDITION (CHE), a TEOSINTE BRANCHED1-CYCLOIDEA-PCF (TCP) transcription factor, to directly repress *CCA1* transcription [41]. Collectively, the action of this set of PRR repressors restricts the expression of *CCA1* and *LHY* to a narrow window around dawn. Each of the PRRs, including TOC1, represses the preceding PRRs, as well as targets distinct sets of clock output genes [36,37,38]. TOC1 also represses genes that encode components of the evening complex (EC), including LUX ARRHYTHMO (LUX), a MYB-like GARP transcription factor [32,33]. The EC consists of LUX or the close LUX homolog, BROTHER OF LUX ARRHYTHMO (BOA, also known as NOX), complexed with EARLY FLOWERING 3 (ELF3) and ELF4 [42,43,44]. The EC is a transcriptional repressor and has been shown to bind to the promoters of *PRR9*, *PRR7*, and *LUX* itself [45,46]. LUX and BOA are not fully redundant; likely two ECs, one including LUX and a second including BOA, share some targets but also regulate distinct gene sets, permitting more nuanced regulation of output genes.

### 2.2. Transcriptional Activation

All the transcriptional regulation described up to this point has been repression, and it has been possible to model the *Arabidopsis* oscillator as a “repressilator” [47]. However, more recently it has become apparent that a number of transcriptional activators play important roles. LIGHT-REGULATED WD1 (LWD1) and LWD2 are transcriptional co-activators recruited to the promoters of several clock genes, including *CCA1*, *PRR9*, *PRR5*, and *TOC1* [48,49]. Recruitment to the promoter and transcriptional activation of *CCA1* is mediated by the interaction of LWD1 and LWD2 with two TCP transcription factors related to CHE, TCP20 and TCP22 [49]. TCP20 transcript cycles with a pre-dawn maximum [50] are consistent with a role in *CCA1* regulation. The transcription factors that are responsible for the recruitment of the LWDs to the promoters of dusk-expressed genes, such as *TOC1,* remain unknown.

Three CCA1/LHY homologs, REVEILLE8 (RVE8), RVE4, and RVE6 provide a second set of transcriptional activators phased later in the day than the TCP/LWD complexes [51,52,53]. These RVEs form complexes with transcriptional coactivators, NIGHT LIGHT-INDUCIBLE AND CLOCK-REGULATED1 (LNK1), and LNK2 to activate expression of *PRR5*, *TOC1*, and *ELF4* [54,55].

Further transcriptional activation of ELF4 is provided by FAR-RED ELONGATED HYPOCOTYL3 (FHY3), FAR-RED IMPAIRED RESPONSE1 (FAR1), and ELONGATED HYPOCOTYL5 (HY5), three transcription factors that are positive regulators of phytochrome A signaling [56]. At least in part, the transcriptional repression activity of CCA1 and LHY derives from their interaction with and inhibition of the transcriptional activation activity of FHY3, FAR1, and HY5 [56].

Recent models of the Arabidopsis clock have begun to incorporate transcriptional activation [57]. It seems likely that additional transcriptional regulators, both positive and negative, of central clock oscillator genes remain to be identified and characterized, further complicating a burgeoning network of interlocked feedback loops.

### 2.3. Chromatin Structure

Activated expression of the critical clock genes *CCA1*, *LHY*, and *TOC1*, is associated with increases in the active chromatin marks trimethylated lysine 4 of histone H3 (H3K4me3) and acetylated lysines 9 and 14 (H3K9/14Ac) [58,59]. The opposing activities of the two sets of Myb transcription factors on *TOC1* expression, with CCA1/LHY repressing and RVE8/6/4 activating, are associated with opposing effects on chromatin structure. CCA1 repression of *TOC1* expression is associated with histone H3 deacetylation of the *TOC1* promoter [60]. Conversely, RVE8 activation of *TOC1* is associated with H3 acetylation [51].

As indicated above, LNK proteins serve as co-activators for the RVEs. The RVE proteins have sequence-specific DNA-binding activity through their Myb domain. A second domain of RVE8, the LCL domain, recruits the LNKs through protein-protein interaction. The LNKs serve to recruit RNA polymerase II and the transcription elongation factor SSRP1 (STRUCTURE-SPECIFIC RECOGNITION PROTEIN1), a component of the transcription elongation FACT complex, to support transcript initiation and elongation of both *TOC1* and *PRR5* [61]. Associated with LNK recruitment is the accumulation of the active chromatin mark, trimethylated lysine 4 of histone H3 (H3K4me3) [61]. Rhythmic changes in H3K4me3 are controlled by the histone methyltransferase SDG2/ATXR3 (SET DOMAIN GROUP2/ARABIDOPSIS TRITHORAX-RELATED3) [62] and the histone demethylase JMJD5/JMJ30 (JUMONJI DOMAIN CONTAINING5/30) [63,64]. *JMJD5/JMJ30* expression is itself repressed by CCA1 and LHY through direct promoter binding [64]. In addition to JMJD5/JMJ30, the histone demethylases LYSINE-SPECIFIC DEMETHYLASE1 (LSD1) AND LSD2, also interact with CCA1/LHY to repress *TOC1* expression. The LSDs are complexed with HISTONE DEACETYLASE6 (HDA6), allowing for coordinated histone demethylation and deacetylation of the *TOC1* promoter [65].

## 3. Post-Transcriptional Regulation Is Pervasive in the Plant Clock

### 3.1. Transcript Stability

Although studies on circadian regulation of gene expression have emphasized transcriptional control, transcript abundance can also be modified through regulated stability. For example, a systematic survey of the *Arabidopsis* transcriptome found that many clock-controlled transcripts have short half-lives [66]. It was subsequently demonstrated that, for some of these, the transcript stability changed over the circadian cycle [67]. The downstream (DST) element was implicated in the degradation of these mRNAs and disruption of the DST pathway altered function of the circadian clock, shown by a lagging phase in the leaf movement rhythm [67]. *CCA1* mRNA is destabilized in the light, likely contributing to normal entrainment to the diurnal light-dark cycle [68].

### 3.2. Alternative Splicing

A second post-transcriptional mechanism of considerable importance to the Arabidopsis circadian clock is alternative splicing (AS) [69]. One particularly well understood example is the auto-regulatory output loop of two *GLYCINE-RICH RNA-BINDING PROTEIN* (*GRP*), genes, *GRP7* (also called *COLD AND CIRCADIAN REGULATED2*, *CCR2*) and *GRP8*. The accumulation of the GRP proteins allows binding to their own transcripts to promote an AS event, resulting in the retention of an intron containing a premature termination codon (PTC), which triggers transcript degradation via nonsense-mediated decay (NMD) [70,71,72,73]. GRP7 and GRP8 regulate stability and AS of additional cycling transcripts [74,75].

Alternative splicing is widespread among *Arabidopsis* transcripts [76], including among clock gene transcripts such as *CCA1*, *LHY*, *RVE8*, *PRRs*, *TOC1*, *ELF3*, and *GIGANTEA* (*GI*) [69,77,78,79,80,81,82,83]. AS events have also been detected in *LUX*, *TIME FOR COFFEE* (*TIC*), and *LOV KELCH PROTEIN2* (*LKP2*) transcripts at elevated temperatures, although their biological significance has not yet been demonstrated [78].

Many components of the splicing machinery have been implicated in the regulation of AS of clock transcripts [84]. For example, the loss of function mutations in *PROTEIN ARGININE METHYLTRANSFERASE5* (*PRMT5*) lengthen the circadian period [85,86]. PRMT5 modifies a number of important splicing machinery components [87], including some SM-LIKE (LSM) proteins that are components of the spliceosomal U6 small nuclear ribonucleoprotein complex. Several *LSM* transcripts cycle and reduced function of several LSM proteins lengthens circadian period in both *Arabidopsis* and humans [88].

The loss of function mutation of *SPLICEOSOMAL TIMEKEEPER LOCUS1* (*STIPL1*), which encodes a component of the complex that controls spliceosome disassembly, results in reduced splicing of many introns. In particular, it results in the retention of intron 3 in the *PRR9* transcript, which leads to a non-functional protein. Consistent with the phenotype of a loss of function *prr9* mutation, *stipl1* confers long circadian period [89].

AS is emerging as a potent mechanism by which clock function is modulated in response to environmental stress, particularly to temperature [90]. Low temperature increases the frequency of intron retention events in *LHY*, *TOC1*, and *PRR7* transcripts that would result in translation into non-functional isoforms [79,81,82]. *CCA1* and *LHY* can be distinguished by their temperature responses, with *LHY* more important than *CCA1* for clock function at higher temperatures, and *CCA1* is more important than *LHY* at lower temperatures [91]. At least in part, this can be explained through temperature-responsive AS [82]. High temperature increases an AS of *CCA1* with the retention of the fourth intron (*CCA1-IR4*), yielding an mRNA isoform containing a PTC and likely subject to NMD [76]. Cold treatment increases the abundance of the functional CCA1 isoform. Cooler temperatures increase the frequency of two AS events in *LHY*: Retention of an intron in the 5’UTR and the inclusion of an additional exon 5a, within the coding sequence, are temperature-responsive [82,92]. The inclusion of exon 5a upon cooling introduces a PTC and is predicted to reduce the levels of functional LHY at lower temperatures. The functional consequences of the retention of the 5’UTR intron are not known, but may include altered translation of the mRNA. The retention of the 5’UTR intron is itself a consequence of temperature-sensitive AS of transcripts encoding several splicing factors, including polypyrimidine tract-binding protein1 (PTB1), PTB2, U2 associated factor 65A (U2AF65A) and suppressor of ABI3-5 (SUA), that contribute to the splicing of the *LHY* pre-mRNA. Thus, a cascade of AS contributes to the reduction of LHY protein at low temperature [82,92].

A number of components of the splicing apparatus have been shown to affect clock function in a temperature-sensitive fashion. For example, mutation of the *SKIP* gene, encoding the spliceosomal component SNW/Ski-interacting Protein, also confers long-period in a temperature-sensitive fashion (the period is lengthened at low, but not at high temperatures). SKIP associates with and affects AS splicing of many transcripts, notably including those of *PRR7* and *PRR9* [81]. In contrast, the mutation of *GEMIN2*, which encodes a spliceosomal snRNP assembly factor, results in an intron retention event in the *TOC1* transcript that reduces TOC1 protein accumulation; the resultant period shortening is most evident at higher temperature [79]. Mutation of SICKLE (SIC), a nuclear protein of unknown function, but implicated in AS, results in temperature-dependent AS of *LHY*, *CCA1*, *PRR7*, and other transcripts, and confers a long period and impaired temperature compensation [93].

### 3.3. Translation

Proteomic analysis has indicated that there is often a failure of protein levels to track transcript abundance [94], suggesting that, either protein synthesis, or stability, or both may be major determinants of gene expression. In the unicellular alga *Acetabularia mediterranea*, circadian rhythms occur at the level of cytoplasmic protein synthesis and persist even when the nucleus has been removed from the cell [95,96]. In the dinoflagellate *Lingulodinium* (formerly *Gonyaulax*) *polyedra*, circadian regulation occurs at the level of translation without circadian cycles in transcript abundance [97,98]. In mammals, the circadian clock regulates the transcription of ribosomal protein mRNAs, ribosomal RNAs, and translation initiation factors as well as their activity [99]. Ribosomal profiling supports the importance of the circadian control of translation in the regulation of gene expression; circadian oscillations in translational efficiency not only affect the protein profiles of genes whose transcripts cycle, but in some cases confer oscillations in protein abundance on genes whose transcripts do not cycle [100].

In *Arabidopsis*, many (30–40%) proteins with cycling protein levels do not have cycling transcript levels and many cycling transcripts encode proteins that do not cycle in abundance, indicating widespread control at the level of translation or of protein stability [94,101,102]. A substantial proportion (~15%) of mRNAs show diel cycles in ribosome loading, and cycling ribosome loading persisted in continuous light, establishing a role of the circadian clock in the regulation of ribosome loading. Several clock genes, including *TOC1*, *LUX*, *GI*, and *PRR5*, show an ~6 h lag between maximal transcript abundance and maximal translation rate, consistent with translational control [101]. Translation of *LHY* mRNA is environmentally responsive and is increased by light [103]. Although the study of circadian regulation of translation and of translational control in modulating clock function is in its infancy, it seems likely that this will prove a fertile ground for future study [104].

### 3.4. Protein Stability

**Figure 2:**
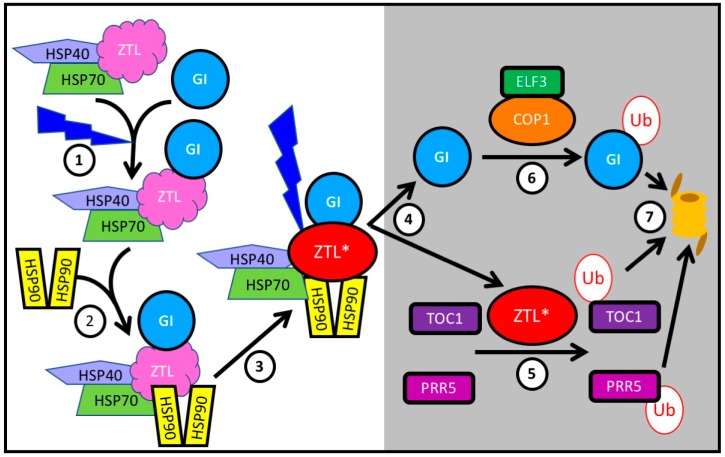
GI is a co-chaperone interacting with HSP90 to facilitate the maturation of ZTL, which is released after dark to ubiquitylate TOC1 and PRR5, targeting them for proteasomal degradation. ① Immature ZTL interacts with HSP70 (and probably HSP40). The recruitment of GI to this complex is facilitated by blue light. ② Subsequently HS90 homodimers are recruited to this complex. ③ GI plays a role as co-chaperone in the maturation of ZTL, here indicated as ZTL*. Blue light stabilizes the interaction of GI with ZTL, sequestering it from its targets TOC1 and PRR5. ④ After dusk, ZTL is released from GI and ⑤ ubiquitylates TOC1 and PRR5, targeting them for proteasomal degradation ⑦. ⑥ Free GI is itself ubiquitylated by a complex of COP1 with ELF3, and ubiquitylated GI is degraded by the proteasome ⑦.

It is abundantly clear that regulated degradation of clock components is crucial to normal clock function. In *Arabidopsis*, three closely related F-box proteins [ZEITLUPE (ZTL), FLAVIN BINDING, KELCH REPEAT F-BOX1 (FKF1), and LOV KELCH PROTEIN 2 (LKP2)] containing blue-light-sensing LOV (Light, Oxygen, and Voltage) domains and Kelch protein-protein interaction domains serve as E3 ubiquitin ligase elements of SCF (Skp-Cullin-F box) complexes and are important stability-determinants for key clock components, including PRR5 and TOC1 [105,106,107]. Although the mRNA abundance of *ZTL* does not cycle, rhythmic abundance of ZTL protein, peaking at dusk, is conferred through a stabilizing interaction with GI, which cycles in abundance at both mRNA and protein levels [108,109]. Immature ZTL initially complexes with HSP70 (and probably HSP40). GI and subsequently HSP90 are recruited to this complex, with GI serving as a protein co-chaperone to enhance the HSP90/HSP70-dependent maturation of ZTL (Figure 2) [110,111,112]. The blue-light dependent interaction of GI with ZTL is destabilized after dark, releasing ZTL from GI and allowing its interaction with its targets; both targets and ZTL are proteasomally degraded [107,108] (Figure 2). The stability of GI protein is itself regulated; the EC component ELF3 protein interacts with the E3-ubiquitin ligase COP1 to ubiquitylate GI, targeting GI for proteasomal degradation at night, when both ELF3 and COP1 are maximally abundant [113] (Figure 2). ZTL is the predominant determinant of PRR5 and TOC1 stability, but both FKF1 and LKP2 interact with TOC1 and PRR5 and loss of function mutations of *fkf1* and *lkp2* enhance the long period phenotype of *ztl* loss of function mutants, indicating that ZTL, FKF1, and LKP2 are functionally redundant and share these and possibly other targets [114,115]. Recently, CHE has been identified as an additional ZTL target [116]. PRR9, PRR7, and PRR3 are related to PRR5 and TOC1 and are proteasomally degraded, but are not ubiquitylated by ZTL [107]. Opposing the ubiquitylation of multiple proteins are a family of deubiquitylating enzymes, two of which, UBP12 and UBP13, are circadian regulated and contribute to period definition, with loss-of-function mutants exhibiting short period [116,117]. Sub-cellular localization also contributes to clock function. GI functions in the nucleus as an activator, and in the cytoplasm as a repressor of *LHY* [118]. Within the nucleus, ELF4 physically interacts with GI to regulate its access to chromatin through sequestration into nuclear bodies [119].

### 3.5. Protein Modification

Phosphorylation plays an important role in all circadian systems [4]. It has been known for many years that the regulatory subunits of the Ser/Thr protein kinase CK2 interact with and phosphorylate CCA1 and LHY; phosphorylation affects dimerization and reduces DNA-binding activity [120,121,122,123]. Simultaneous loss of the three catalytic nuclear-localized CK2 subunits lengthens circadian period, confirming the importance of CK2 activity for clock function [124]. Elevated temperature stimulates both CCA1 binding to target promoters and CK2 phosphorylation of CCA1, which reduces its DNA-binding activity; these two opposing activities counter-balance and contribute to temperature compensation [123]. CK2 activity itself is clock-regulated; the regulatory CKB4 subunit is phosphorylated, ubiquitylated, and degraded by the proteasome during the day [125].

TOC1 and the PRRs also exhibit time-of-day specific phosphorylation, although the consequences of this phosphorylation are complex. Phosphorylation of TOC1 and PRR5 increases their affinities for ZTL, thereby promoting their ubiquitylation and degradation [107]. However, the interaction of TOC1 and PRR5 promotes the nuclear accumulation and phosphorylation of TOC1 [115]; nuclear TOC1 is sequestered from cytoplasmic ZTL and therefore stabilized [107]. Phosphorylation of PRR3 and TOC1 enhances their interaction, which sequesters TOC1 from ZTL, thereby promoting its stability [107,115,126]. Although the kinase(s) responsible for PRR phosphorylation are not known, phosphoproteomic analysis has identified a number of candidates [127]. This study showed that there are extensive circadian changes in the phosphorylation status of many proteins, including among likely regulatory molecules such as the clock components ELF4 and PRR3, multiple transcription factors, and protein kinases. The phosphorylation of ELF4 enhances its interaction with ELF3, a key partner in the EC, and the mutational replacement of a key phosphorylated residue on ELF4, with a non-phosphorylatable residue (S45L) lengthens the circadian period and enhances temperature compensation, especially at higher temperatures [127].

## 4. Multiple Tissue- and Organ-Specific Clocks

Studies in protoplasts support the presence of an autonomous clock in each plant cell [128,129,130]. To what extent are these cell-autonomous clocks coupled? The two cotyledons and the shoot apex can be entrained independently, suggesting no, or very weak, coupling [131]. However, other studies in *Kalanchoe*, *Arabidopsis*, and *Lemna* have suggested some degree of coupling among cells [132,133,134,135]. In contrast to the weak coupling seen in leaves, evidence suggests that clocks in the cells of shoot apex are tightly coupled [136]. Excised shoot apices retain robust synchrony for several days. However, cultures of dissociated protoplasts derived from the shoot apex rapidly lose synchrony, and dilution of the protoplasts accelerates the loss of synchrony. This demonstrates a crucial role for intercellular communication (coupling) in the maintenance of coherent rhythms among cells of the shoot apex, although the molecular mechanisms of coupling are not known at this time [136].

It is well-established that mammals have a hierarchical clock organization with a central clock in the suprachiasmatic nucleus (SCN), providing synchronization to peripheral clocks distributed in the various tissues of the body [137]. Evidence is accumulating in support of the existence of multiple tissue-specific clocks in plants, although the hierarchical nature of their organization is not yet well-understood [138]. The first evidence in support of multiple tissue-specific clocks came with the observation in bean that the circadian periods of leaf movement and stomatal conductance differ [139]. Similar differences in the periods of rhythmic cytosolic Ca^2+^ levels and *LHCB* promoter activity were seen in tobacco [140]. In *Arabidopsis*, it was established that the periods of the transcription of different genes differed [141,142] and that different genes were differentially responsive to light versus thermal entrainment [143], again consistent with distinct clocks in distinct tissues or organs.

The existence of tissue-specific clocks in the mesophyll, epidermis, and vasculature, was confirmed through transcriptomic analysis of isolated tissues [144] and subsequently through tissue-specific clock disruption via the use of tissue-specific promoters driving overexpression of CCA1 or TOC1, which results in arrhythmia [145]. Arrhythmia in the phloem companion cells disrupted photoperiodic induction of flowering but had no effect on hypocotyl elongation. In contrast, arrhythmia in the epidermis had no effect on flowering, but instead disrupted the day-length control of hypocotyl elongation. Therefore, the leaf vascular circadian clock is critical to photoperiodic flower induction and the epidermal clock controls hypocotyl elongation [145].

The existence of tissue-specific clocks also raises the question of their potential hierarchical organization. The leaf vascular circadian clock influences the mesophyll clock, but perturbation of the mesophyll clock has no effect on vascular clock function [144]. Signals from the shoot, likely including photosynthetic signals, have been shown to affect rhythmicity in the roots [146]. Ablation of the shoot apex results in rapid dampening of root rhythms, indicating that shoot apex to root signaling is crucial for the maintenance of robust root rhythms [136]. However, perturbation of the clock in the shoot apex does not affect photoperiodic flowering or hypocotyl elongation [144,145], demonstrating that the shoot apex does not house a master oscillator analogous to the SCN clock.

Several outstanding issues remain to be resolved. First and foremost, do the clocks in different tissues differ in terms of composition and function? For example, PRR3 has been suggested to be specific to the leaf vasculature clock [126]. More recently, the shoot apex clock has been shown to persist in the absence of LUX function, whereas *lux* loss of function mutants are arrhythmic in other tissues [136,147]. It is reasonable to hypothesize that the robust coupling at the shoot apex confers persistent rhythmicity in the absence of LUX, but perhaps other components have been recruited to replace LUX function in the shoot apex clock. Second, how is coupling accomplished among cells within a tissue and between clocks in distinct tissues? The long distance signaling between the clocks of the shoot apex and the root is not well understood in mechanistic detail. Multiple mobile signals, including auxin, photosynthates such as sucrose, and the transcription factor HY5 have been shown to move from the shoot to the root [148,149]. Auxins have been shown to regulate both amplitude and precision of circadian rhythms [150], although a role in shoot-to-root clock signaling remains speculative. HY5 is known to regulate *ELF4* expression (Figure 1) [56], although the relevance of HY5 translocated from the shoot to the root to root clock function remains to be established. Sucrose entrains the clock via a sugar-responsive kinase that modulates the activity of a transcription factor that regulates *PRR7* expression [151,152] and seems quite likely to play a role in regulation of the root clock, but whether sucrose contributes to the signaling between the shoot and root clocks is not known. Finally, light impinging on the above-ground parts of *Arabidopsis* plants is piped into the roots where it can both entrain the clock and influence period length directly, independent of mobile shoot-to-root signals of plant origin [153].

## 5. Concluding Remarks

Plant circadian clocks share a common architecture with clocks of all eukaryotes: Interlocked negative feedback loops [1,154]. Like other eukaryotic clocks, plant clocks employ a diverse array of transcriptional and post-transcriptional regulatory mechanisms to establish a robust oscillation that is resilient in the face of environmental fluctuations, yet responsive to environmental time cues. However, plant clocks are distinguished from other eukaryotic clocks by their complexity. During the evolution of the green lineage, the circadian clock has become increasingly complex, and the common angiosperm ancestor of monocots and eudicots had components sufficient to construct a circadian clock consisting of multiple interlocked feedback loops [155,156]. It seems reasonable that repeated whole genome duplication (WGD) events that have occurred during the evolution of the green lineage have facilitated that increase in complexity [157,158]. Experimental evidence indicates that the complexity of the plant circadian oscillator enhances the maintenance of robust rhythms across a broad range of environmental conditions [159,160]. Impaired circadian function reduces plant growth and fitness, offering the hypothesis that optimizing circadian function will enhance crop productivity, particularly in crops grown over broad latitudinal ranges. This hypothesis has found recent support in studies identifying clock components as domestication genes [161,162]. A greater refinement of our understanding of the circadian clock mechanism will inform efforts to manipulate the circadian clock towards the goal of crop improvement and the enhancement of agricultural productivity.
